# Subcutaneous chondromyxoid fibroma with a novel *PNISR::GRM1* fusion—report of a primary soft tissue tumour without connection to an underlying bone

**DOI:** 10.1007/s00428-023-03519-4

**Published:** 2023-02-21

**Authors:** Andrej Zupan, Vladka Salapura, Daja Šekoranja, Jože Pižem

**Affiliations:** 1 Institute of Pathology, Faculty of Medicine, University of Ljubljana, Korytkova 2, 1000 Ljubljana, Slovenia; 2 Institute of Radiology, University Medical Centre Ljubljana, Zaloška 7, 1000 Ljubljana, Slovenia; 3 Faculty of Medicine, University of Ljubljana, Vrazov trg 2, 1000 Ljubljana, Slovenia

**Keywords:** Chondromyxoid fibroma, Extraosseous, *GRM1*, *MEF2A::ARHGAP36*, *PNISR::GRM1*, Subcutaneous

## Abstract

Chondromyxoid fibroma (CMF) is a rare benign bone tumour. While CMF located entirely on the surface of a bone (i.e. juxtacortical CMF) has been well characterised, CMF has not so far been convincingly documented to arise in soft tissues without connection to an underlying bone.

We report a subcutaneous CMF in a 34-year-old male, located on the distal medial aspect of the right thigh without any connection with the femur. The tumour measured 15 mm, it was well-circumscribed and displayed typical morphological features of a CMF. At the periphery, there was a small area of metaplastic bone. Immunohistochemically, the tumour cells were diffusely positive for smooth muscle actin and GRM1, and negative for S100 protein, desmin and cytokeratin AE1AE3. Whole transcriptome sequencing revealed a novel *PNISR*::*GRM1* gene fusion.

Our case indicates that CMF should be included in the differential diagnosis of soft tissue (including subcutaneous) tumours composed of spindle/ovoid cells, with a lobular architecture and chondromyxoid matrix. The diagnosis of CMF arising in soft tissues can be confirmed by identifying a *GRM1* gene fusion or GRM1 expression by immunohistochemistry.

**Supplementary Information:**

The online version contains supplementary material available at 10.1007/s00428-023-03519-4.

## Introduction

Chondromyxoid fibroma (CMF) is a rare benign bone tumour accounting for 1% of all primary bone tumours [1]. It is most frequently located in the metaphysis of long bones (predominantly the proximal tibia) and typically affects adolescents and young adults but can occur in practically any bone and in a wide age range [2, 3]. It is usually a well-demarcated, eccentric intraosseous lesion but can expand the bone and extend into the adjacent soft tissue. Rare CMF are located entirely on the surface of a bone (i.e. juxtacortical CMF) [4–6].

Microscopically, CMF is a lobular neoplasm with a characteristic zonal pattern—a more cellular periphery in which spindle cells predominate and less cellular centres of the lobules composed of stellate and chondroid-appearing cells within the chondromyxoid matrix. Calcifications are present in about one-third of cases; they are particularly common in craniofacial tumours and rare in children [1–3]. Genetically, CMF is characterised by a glutamate receptor *GRM1* gene rearrangement, which places the *GRM1* gene under the influence of strongly active gene promoters, including *COL12A1*, *MEF2A* and *BCLAF1* [7]. This results in a significant overexpression of the GRM1 protein, which can be detected by immunohistochemistry [3, 7]. In a recent study, GRM1 was positive in 97% of CMF but was negative in its morphological mimics [3].

To the best of our knowledge, a pure soft tissue CMF, not arising from a bone or a bone surface, has not been convincingly documented in the literature [2, 5]. In the largest published series of 278 CMF, Wu et al. mentioned two cases (not included in the series), which appeared to be primarily located in soft tissues without any bone involvement, one in the soft palate and one between the anus and vagina. However, a detailed description was not provided and the diagnosis was not supported by molecular studies [2]. Because CMF is not expected in soft tissues (unrelated to a bone), it is possible for a soft tissue CMF to remain under-recognised. We report a purely subcutaneous CMF harbouring a novel *PNISR::GRM1* gene fusion, adding CMF in the differential diagnosis of tumours arising in superficial soft tissues.

## Materials and methods

### Immunohistochemistry

The following immunohistochemical stains were performed: smooth muscle actin (clone 1A4, dilution 1:100, Cell Marque, Rocklin, CA), desmin (clone D33, dilution 1:20, DAKO, Glostrup, Denmark), S100 protein (polyclonal, dilution 1:1000, DAKO, Glostrup, Denmark), cytokeratin (clone AE1, AE3, dilution 1:50, Novocastra–Leica Biosystems, Newcastle Upon Tyne, UK), CD34 (clone QBEnd-10, dilution 1:20, DAKO, Glostrup, Denmark), ERG (clone 9FY, dilution 1:40, Biocare Medical, CA) and GRM1 (clone JM11-61, Invitrogen–Thermo Fisher Scientific, dilution 1:100, Walthman, MA). Immunohistochemical staining was performed in automatic immunostainers Benchmark XT or Benchmark Ultra (desmin, GRM1), (Ventana Medical Systems Inc., Tucson, AZ).

### Molecular methods

RNA was extracted from formalin-fixed and paraffin-embedded (FFPE) tumour tissue using a Maxwell FFPE RNA kit (Promega, Madison, WI). RNA concentration and purity were measured using a Qubit 3.0 Fluorometer (Thermo Fisher Scientific, Waltham, MA). Libraries were prepared according to the protocol of the Qiaseq Stranded RNA Library Kit (Qiagen, Hilden, Germany), and the library quality was assessed using a Bioanalyzer 2100 (Agilent Technologies, Santa Clara, CA). Whole transcriptome sequencing was performed with a paired-end 150 bp read length. Detection of fusion transcripts was performed using STAR-Fusion (1.10.1) software [8] mapped to the reference genome (hg38) using STAR aligner [9]. A supervised analysis of fusion predictions was performed using FusionInspector (2.6.0) software. The gene expression was evaluated using the quantMode option in the STAR aligner. Validation of fusion transcripts was performed with RT-PCR and Sanger sequencing (Supplementary Table 1) using standard BigDye 3.1 chemistry (Thermo Fisher Scientific, Waltham, MA). Visualisations of fusion transcripts and the creation of Sashimi Plots were performed with IGV 2.12.3 software [10].

## Results

### Clinical and imaging findings

**Fig. 1:**
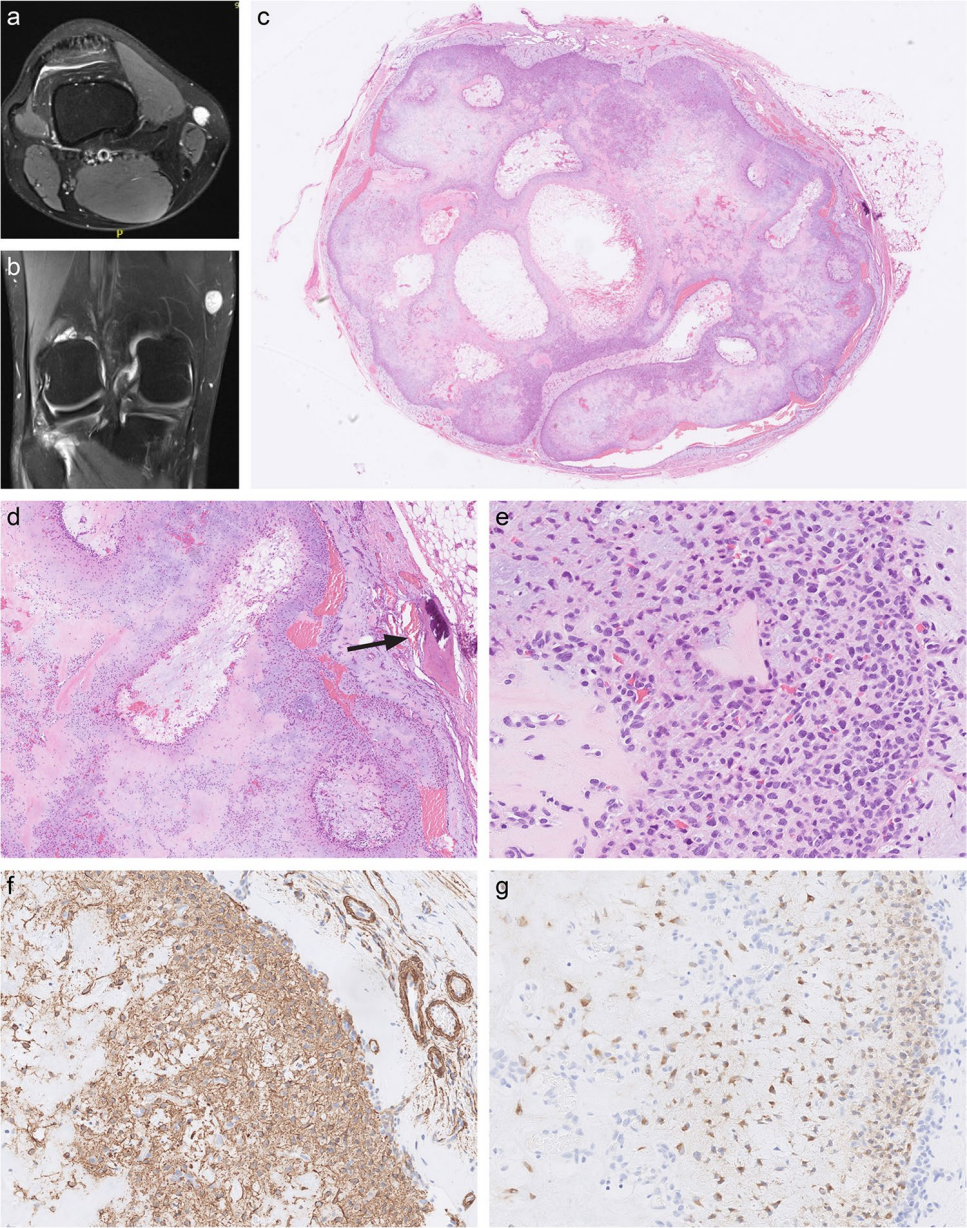
MR imaging of the right knee (**a**, **b**). An axial proton density FS image shows a well-defined, oval tumour in the subcutis, with mostly hyperintense signal. It is located on the distal medial aspect of the right thigh at the level of the distal femoral metaphysis. On sagittal proton density FS image (**b**), the tumour exhibits a heterogeneous appearance with small, separate areas of hyperintense signal surrounded by tissue of isointense signal and with hyperintense margin. Histopathological features (**c**–**g**). A well-circumscribed, lobulated subcutaneous tumour with focal bone metaplasia at the periphery (**d**, arrow), increased cellularity at the periphery of the lobules and chondromyxoid matrix toward the centre of the lobules. Diffuse positivity for smooth muscle actin (**f**) and diffuse cytoplasmic positivity for GRM1 (**g**) in the tumour cells (note the absence of positivity in the endothelial cells adjacent to and within the tumour lobule)

A 34-year-old male presented with a 15 mm subcutaneous tumour on the distal medial aspect of the right thigh. MR imaging (Figs. 1a and b) showed a well-delineated tumour without connection with the femur. The tumour was excised. There was no evidence of disease 22 months after a marginal excision.

### Pathological findings

The excised tumour measured 15 × 15 × 10 mm and was brown-white and firm on the cut surface. Microscopically, the tumour was well circumscribed and lobulated. The lobules were more cellular at the periphery, where ovoid and spindle cells predominated. Towards the centre of the lobules, the tumour was less cellular, composed of spindled, stellate and chondroid-appearing cells in an abundant chondromyxoid matrix (Figs. 1 c–e). There were no calcifications, significant cytological atypia, or mitotic activity. A small area of morphologically benign metaplastic bone at the periphery of the tumour was present (Fig. 1d). The tumour stroma was well vascularised, in particular at the periphery of the lobules. The tumour cells were diffusely positive for smooth muscle actin and GRM1 (in > 95% of the neoplastic cells) [3] (Figs. 1f and g), focally positive for CD34 and negative for S100 protein, desmin, cytokeratin AE1AE3 and ERG. Based on the morphology and immunophenotype (without the GRM1 stain, which was not available at the time of original diagnosis), the case was originally signed out as morphologically consistent with a CMF.

### Molecular findings

The whole transcriptome analysis identified two in-frame fusions (Table 1, Supplementary Figure 1), which were validated by RT-PCR and Sanger sequencing—a *PNISR* (5' UTR region)*::GRM1* (5' upstream region) fusion, producing a full-length GRM1 protein, and a *MEF2A* (3' UTR region)*::ARHGAP36* (exon2) fusion. The latter resulted in a deletion of 31 amino acids in the N terminal region of the ARHGAP36 protein while retaining the in-frame coding potential of the *ARHGAP36* gene.

**Table 1:** Detected fusion transcripts with descriptive data as obtained with the STAR-Fusion algorithm

Fusion	*PNISR::GRM1*	*MEF2A::ARHGAP36*
Junction reads	7	65
Left gene	PNISR^ENSG00000132424.17	MEF2A^ENSG00000068305.17
Left breakpoint	chr6:99425215 (-)	chr15:99598511 ( +)
Right gene	GRM1^ENSG00000152822.14	ARHGAP36^ENSG00000147256.12
Right breakpoint	chr6:146027648 ( +)	chrX:131081760 ( +)
Raw reads counts per gene	2014 (PNISR), 935 (GRM1)	4683 (MEF2A),3815 (ARHGAP36)
Fusion type	Intra-chromosomal (chr6-chr6; 46.60 Mb)	Inter-chromosomal (chr15-chrX)

## Discussion

We present a subcutaneous CMF with a novel *PNISR::GRM1* fusion. Although a soft tissue extension of an intraosseous CMF is well recognised and CMF may occasionally arise on the surface of a bone (i.e. juxtacortical CMF) [4–6], to the best of our knowledge CMF has not been convincingly documented to arise in soft tissues without connection to an underlying bone.

In addition to the relatively characteristic morphological appearance, the diagnosis in our case was further substantiated by the identification of a *GRM1* gene fusion and immunohistochemical positivity for GRM1. Apart from GRM1 positivity, the immunophenotype of CMF is relatively non-specific. It is consistently positive for smooth muscle actin, variably positive for S100 protein (in 8–86% of cases according to different series) and EMA, and negative for desmin and cytokeratins [4, 11]. A recent study showed that GRM1 immunohistochemistry may be a useful ancillary technique to confirm the diagnosis of CMF and to differentiate it from morphological mimics, since it is not expressed in other bone tumours, which may be considered in the differential diagnosis with CMF [3].

Previous studies have identified activation of the *GRM1* gene as a specific driver event for the development of CMF [7]. CMF is characterised by fusions involving the *GRM1* gene, which are present in the vast majority of CMF. Several upstream partner genes have been identified, including *COL12A1*, *MEF2A* and *BCLAF1* [3, 7]. Using whole transcriptome sequencing, we identified a novel *PNISR::GRM1* fusion, along with the additional *MEF2A::ARHGAP36* fusion. The *PNISR* gene encodes the PNISR protein, which is part of protein complexes in corneal epithelial cells. The exact function of the PNISR protein is unknown [12]. The *PNISR* gene acts as a partner gene in the promoter-swapping mechanism between *PNISR* and *GRM1*, presumably leading to upregulated *GRM1* gene expression [7]. The significance of the *MEF2A::ARHGAP36* fusion remains unknown and, to our knowledge, has not been previously documented (Mitelman Database, https://mitelmandatabase.isb-cgc.org). *MEF2A* has been reported as a mediator in bone formation and as an upstream partner gene of the *GRM1* gene in CMF [7, 13]. *ARHGAP36* encodes an ARHGAP36 protein, a member of the Rho GTPase-activating protein family involved in spinal cord development and tumorigenesis [14]. Previous studies revealed complex chromosomal rearrangements in CMF, suggesting a high probability of stochastic fusion events, which could explain the presence of various fusions [15, 16]. In this context, the *MEF2A::ARHGAP36* could be a passenger fusion, but further studies are needed to reveal its potential role in CMF.

The differential diagnosis of CMF located in soft tissues includes other tumours with a chondromyxoid or fibromyxoid matrix, including soft tissue chondroma, myoepithelial tumours, ossifying fibromyxoid tumour and phosphaturic mesenchymal tumour [3, 4, 17–20]. Soft tissue chondromas, especially those with chondroblastoma-like features or an extensive myxoid matrix, may closely mimic CMF [17]. Soft tissue chondroma most frequently arises on the hands and feet and may harbour a *FN1::FGFR1/2* fusion [18]. Myoepithelial tumours may morphologically closely resemble CMF. In contrast to CMF, they express cytokeratins and GFAP, do not express GRM1 and harbour a *EWSR1* gene fusion in more than half of the cases [3, 4]. Ossifying fibromyxoid tumour is a lobulated neoplasm with a frequently present bony shell at the periphery (similar to our case of CMF) and sometimes a prominent myxoid or chondroid matrix. In contrast to CMF, ossifying fibromyxoid tumour does not usually show a zonal pattern and there is a uniform cell–cell spacing. S100 protein is expressed in about two-thirds and desmin in half of cases, while smooth muscle actin is only rarely expressed. Ossifying fibromyxoid tumour is in most cases characterised by various gene fusions, most frequently involving the *PHF1* gene [19]. Most phosphaturic mesenchymal tumours are associated with tumour-induced osteomalacia. They are characterised by the expression of FGF23 and harbour *FN1::FGFR1* or *FN1::FGF1* fusions [20].

In conclusion, we report the first molecularly confirmed case of subcutaneous CMF without association with an underlying bone. CMF should be included in the differential diagnosis of a soft tissue tumour with a chondromyxoid stroma. Key morphological features in favour of a CMF are a lobulated architecture and characteristic zonal pattern. Expression of GRM1 detected by immunohistochemistry or demonstration of a *GRM1* gene fusion may assist in the recognition of extraosseous CMF and confirmation of the diagnosis.

## Supplementary Information

**Additional file 1: Supplementary table 1.** Sequences of primers used for fusion transcript validation.

**Additional file 2: Supplementary figure 1.** Sashimi plot of the junction reads. A. Junction reads of the PNISR and GRM1 genes. B. Fusion transcript junction of PNISR::GRM1. C. Gene annotation track of PNISR and GRM1. D. Junction reads of the MEF2A and ARHGAP36 genes. E. Fusion transcript junction of MEF2A::ARHGAP36; F. Gene annotation track of MEF2A and ARHGAP36.

## Data Availability

All data generated or analysed during this study are included in this published article.
