# Supplementary material for: Subcutaneous chondromyxoid fibroma with a novel PNISR::GRM1 fusion—report of a primary soft tissue tumour without connection to an underlying bone
Source: Virchows Arch. 2023 Feb 21;482(5):917–21. doi: 10.1007/s00428-023-03519-4 (PMC10156755; doi:10.1007/s00428-023-03519-4)
Supplement: Supplementary file 1 — Additional file 1: Supplementary table 1. Sequences of primers used for fusion transcript validation. [file 428_2023_3519_MOESM1_ESM.docx]

Supplementary table 1. Sequences of primers used for fusion transcript validation.

| Fusion transcript | Primer sequence | Product size |
| --- | --- | --- |
| *PNISR::GRM1* | Forward:  GTTCCCGAAGAAGTAGAAGCA | 158 bp |
|  | Reverse:  CAGACGCCTCAGCTCTAATC |  |
| *MEF2A::ARHGAP36* | Forward:  TCTGCAAGGATCATATCTAAGTGC | 127 bp |
|  | Reverse:  AGCTCAGAGAGGCTGTGTAT |  |
