# Supplementary material for: Subcutaneous chondromyxoid fibroma with a novel PNISR::GRM1 fusion—report of a primary soft tissue tumour without connection to an underlying bone
Source: Virchows Arch. 2023 Feb 21;482(5):917–21. doi: 10.1007/s00428-023-03519-4 (PMC10156755; doi:10.1007/s00428-023-03519-4)
Supplement: Supplementary file 2 — Additional file 2: Supplementary figure 1. Sashimi plot of the junction reads. A. Junction reads of the PNISR and GRM1 genes. B. Fusion transcript junction of PNISR::GRM1. C. Gene annotation track of PNISR and GRM1. D. Junction reads of the MEF2A and ARHGAP36 genes. E. Fusion transcript junction of MEF2A::ARHGAP36; F. Gene annotation track of MEF2A and ARHGAP36. [file 428_2023_3519_MOESM2_ESM.docx]

**
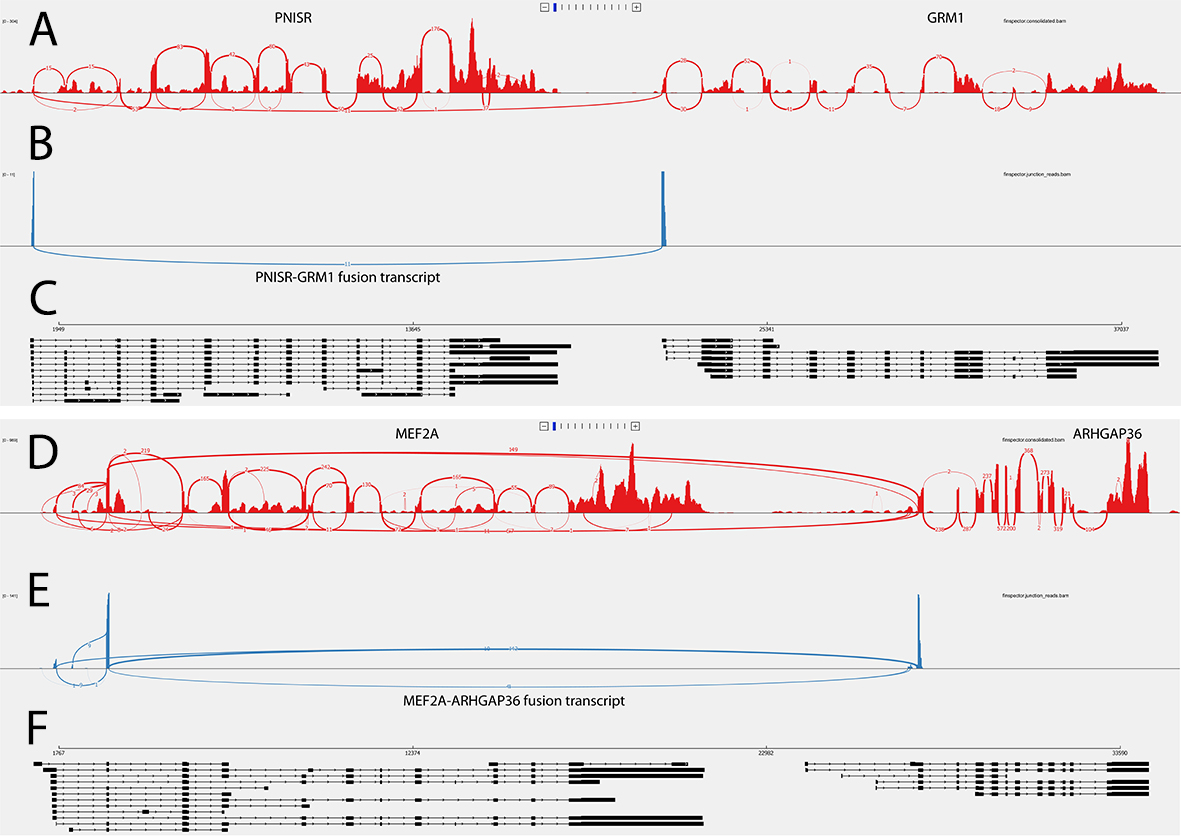
**

Supplementary figure 1. Sashimi plot of the junction reads. A. Junction reads of the *PNISR* and *GRM1* genes. B. Fusion transcript junction of *PNISR::GRM1*. C. Gene annotation track of *PNISR* and *GRM*1. D. Junction reads of the *MEF2A* and *ARHGAP36* genes. E. Fusion transcript junction of *MEF2A::ARHGAP36*; F. Gene annotation track of *MEF2A* and *ARHGAP36*.
